# The radiation- and chemo-sensitizing capacity of diclofenac can be predicted by a decreased lactate metabolism and stress response

**DOI:** 10.1186/s13014-024-02399-5

**Published:** 2024-01-16

**Authors:** Melissa Schwab, Ali Bashiri Dezfouli, Mohammad Khosravi, Bayan Alkotub, Lisa Bauer, Mohammad Javed Tahmasebi Birgani, Gabriele Multhoff

**Affiliations:** 1 Radiation Immuno-Oncology Group, Center for Translational Cancer Research (TranslaTUM), TUM School of Medicine and Health, Klinikum rechts der Isar, Technical University of Munich (TUM), Munich, Germany; 2 Department of Otolaryngology, Head and Neck Surgery, TUM School of Medicine and Health, Klinikum rechts der Isar, Technical University of Munich (TUM), Munich, Germany; 3 Department of Pathobiology, Faculty of Veterinary Medicine, Shahid Chamran University of Ahvaz, Ahvaz, Iran; 4 Institute of Biological and Medical Imaging (IBMI), Helmholtz Zentrum München, Neuherberg, Germany; 5 Department of Radiotherapy and Oncology, Golestan Hospital, Ahvaz Jundi Shapour University of Medical Sciences, Ahvaz, Iran; 6 Department of Radiation Oncology, TUM School of Medicine and Health, Klinikum rechts der Isar, Technical University of Munich (TUM), Munich, Germany

**Keywords:** Radiation sensitization, Diclofenac, Tumor metabolism, Stress response

## Abstract

**Background:**

An enhanced aerobic glycolysis (“Warburg effect”) associated with an increase in lactic acid in the tumor microenvironment contributes to tumor aggressiveness and resistance to radiation and chemotherapy. We investigated the radiation- and chemo-sensitizing effects of the nonsteroidal anti-inflammatory drug (NSAID) diclofenac in different cancer cell types.

**Methods:**

The effects of a non-lethal concentration of diclofenac was investigated on c-MYC and Lactate Dehydrogenase (LDH) protein expression/activity and the Heat shock Protein (HSP)/stress response in human colorectal (LS174T, LoVo), lung (A549), breast (MDA-MB-231) and pancreatic (COLO357) carcinoma cells. Radiation- and chemo-sensitization of diclofenac was determined using clonogenic cell survival assays and a murine xenograft tumor model.

**Results:**

A non-lethal concentration of diclofenac decreases c-MYC protein expression and LDH activity, reduces cytosolic Heat Shock Factor 1 (HSF1), Hsp70 and Hsp27 levels and membrane Hsp70 positivity in LS174T and LoVo colorectal cancer cells, but not in A549 lung carcinoma cells, MDA-MB-231 breast cancer cells and COLO357 pancreatic adenocarcinoma cells. The impaired lactate metabolism and stress response in diclofenac-sensitive colorectal cancer cells was associated with a significantly increased sensitivity to radiation and 5Fluorouracil in vitro, and in a human colorectal cancer xenograft mouse model diclofenac causes radiosensitization.

**Conclusion:**

These findings suggest that a decrease in the LDH activity and/or stress response upon diclofenac treatment predicts its radiation/chemo-sensitizing capacity.

**Supplementary Information:**

The online version contains supplementary material available at 10.1186/s13014-024-02399-5.

## Background

The nonsteroidal anti-inflammatory drug (NSAID) diclofenac negatively affects tumor growth in vitro and in vivo primarily by its inhibitory effect on Cyclooxygenases COX1 and COX2 [1], and also by COX-independent effects associated with a reduced c-MYC expression causing a decreased glucose uptake, lactate production and glutaminolysis [2–4]. Many fast-growing solid cancer cells compensate their heightened energy demand by an elevated aerobic glycolysis also termed “Warburg effect” [5] and a reprogramming of their glucose metabolism. The c-MYC-induced upregulation of relevant glycolytic enzymes such as Glucose Transporter 1 (GLUT1) and Lactate Dehydrogenase A (LDHA) [6, 7] results in an enhanced uptake and conversion of glucose into the oncometabolite pyruvate followed by a LDHA mediated increase in lactic acid and acidification of the tumor microenvironment (TME) [8]. High lactate levels and acidosis are associated with a more aggressive tumor phenotype, an increased risk for metastatic spread, tumor recurrence, immunosuppression and therapy resistance [9, 10]. A downregulation of LDHA activity results in decreased lactate levels in the TME and thereby attenuates tumor progression in preclinical models [11], and restores anti-tumor immune cell functions [12]. In addition to LDHA, Lactate Dehydrogenase B (LDHB), the converting enzyme of lactate to pyruvate [13–15], as well as members of Heat Shock (“stress”) Protein (HSP) families with anti-apoptotic properties are frequently overexpressed in a broad range of different cancer cell types including breast cancer, colorectal carcinoma, lung and pancreatic cancer cells [16]. Increased HSP levels contribute to poor prognosis and therapy resistance [16, 17]. Recently, a link between the lactate metabolism and the stress response has been reported for cancer cells [18]. An *LDHA/B* double knockout significantly reduced the expression of the transcription factor Heat Shock Factor 1 (HSF1) and the major molecular chaperones Hsp90, Hsp70 and Hsp27, and thereby increases the sensitivity towards ionizing irradiation [18]. Although more than 50% of all solid tumors are treated with ionizing radiation and/or chemotherapy, normal tissue toxicity and therapy resistance of tumor cells can limit the clinical success [19]. Therefore, Lactate Dehydrogenase (LDH) inhibition might provide a promising strategy to break therapy (radiation/chemotherapy) resistance by addressing the influence of both, the stress response [20] and lactate metabolism [21]. However, most currently available LDH inhibitors are not suitable for clinical use due to their low in vivo stability and normal tissue toxicity at higher concentrations [22]. In addition to its anti-inflammatory, analgesic, and antipyretic activities [23], the clinically approved compound diclofenac has been shown to inhibit lactate formation and the release of lactate into the TME [2, 4]. Therefore, this study investigated the effects of a tolerable and non-lethal concentration of diclofenac on LDH activity and the stress response in the context of therapy resistance to radiation and chemotherapy in different tumor cell types including LS174T and LoVo colorectal adenocarcinoma cells, A549 lung cancer cells, MDA-MB-231 breast cancer cells and COLO357 pancreatic adenocarcinoma cells. Colorectal cancer cell lines were chosen because previously we have demonstrated that a genetic inhibition of *LDHA* and *LDHB* in LS174T cells impairs the stress response and causes radiation sensitization [18]. Since a gene knockout is difficult to translate into clinical practice, herein, we investigated whether the clinically approved drug diclofenac can phenocopy these effects. A potential chemosensitizing effect of diclofenac was analyzed in combination with 5-Fluorouracil (5-FU) because patients with advanced colon cancer are treated with this chemotherapeutic agent [24]. Lung, breast and pancreatic carcinoma cells were included in the study because these tumor types are frequently treated with radiation [25], and we wanted to determine whether the radiosensitizing effect of diclofenac can be observed in different tumor types.

## Methods

### Cells and cell culture

The LoVo human colorectal adenocarcinoma cell line (ATCC® CCL-229™), the MDA-MB-231 triple-negative human breast adenocarcinoma cell line (ATCC® HTB-26™) and the COLO357 human pancreatic adenocarcinoma cell line were cultured in Roswell Park Memorial Institute (RPMI)-1640 Medium (Sigma-Aldrich/Merck, Darmstadt, Germany). The A549 human lung carcinoma cell line (ATCC® CCL-185™) and the LS174T human colorectal adenocarcinoma cell line (ATCC® CL-188™) were cultured in high glucose Dulbecco`s Eagle`s Minimum Essential Medium (DMEM) (Sigma-Aldrich/Merck). All media were supplemented with 10% v/v heat-inactivated fetal bovine serum (FBS) (Sigma-Aldrich/Merck), 1% antibiotics (10,000 IU/mL penicillin, 10 mg/mL streptomycin, Sigma-Aldrich/Merck), 2 mM L-glutamine (Sigma-Aldrich/Merck) and 1 mM sodium pyruvate (Sigma-Aldrich/Merck). Cells are routinely checked for mycoplasma contamination and only mycoplasma-negative cells were used. All human cell lines were determined as rodent cell free and the genetic identity was authenticated by DNA profiling using 17 different highly polymorphic short tandem repeat loci (Leibniz-Institut DSMZ, Braunschweig, Germany).

### Reagents and treatment

The sodium salt of diclofenac was dissolved in water for all in vitro (Euro OTC Pharma GmbH, Bönen, Germany) and in vivo (Novartis Pharma GmbH, Basel, Switzerland) experiments. Tumor cells were incubated with different concentrations of diclofenac for 48 h if not indicated otherwise. A stock solution (10 mg/mL) of 5-Fluorouracil (5-FU, Sigma-Aldrich/Merck) was prepared in dimethyl sulfoxide (DMSO, Sigma-Aldrich/Merck) and further diluted in phosphate buffered saline (PBS) (Sigma-Aldrich/Merck). Cells were incubated with 1 µM (A549, MDA-MB-231) or 5 µM (LS174T, LoVo) 5-FU for 48 h. Control cells were incubated with the respective amount of the same diluent as a vehicle.

### Cell proliferation

Cell proliferation was measured using a Sigma-Aldrich/Merck Cell Counting Kit 8 (CCK 8), according to the manufacturer’s protocol.

### Lactate dehydrogenase activity measurements

LDH activity was determined using the Lactate Dehydrogenase Activity kit (Sigma-Aldrich/Merck) following the manufacturer’s protocol.

### Western blot analysis

Cells were lysed and processed for Western blotting, as described elsewhere [18] using the following primary and secondary antibodies: anti-HSF1 (1:1,000, ADI-SPA-901-D, Enzo Life Sciences, Farmingdale, NY, USA), anti-Hsp27 (1:1,000, NBP2-32972, Novus Biologicals, Centennial, CO, USA), anti-Hsp70 (1:500, cmHsp70.1, IgG1, multimmune GmbH, Munich, Germany), anti-c-MYC (1:1,000, 5605 S, Cell Signaling Technology, Danvers, MA, USA), anti-LDHA (1:2,000, NBP1-48336, Novus Biologicals, Centennial, CO, USA), anti-LDHB (1:2,000, NBP2-53421, Novus Biologicals, Centennial, CO, USA), antißActin (1:10,000, A2228, Sigma-Aldrich/Merck), HRP-conjugated rabbit anti-mouse immunoglobulins (1:2,000, P0260, Dako-Agilent, Santa Clara, CA, USA) and HRP-conjugated swine anti-rabbit immunoglobulins (1:1,000, P0217, Dako-Agilent). Western blot signals were quantified using the Fiji software [26].

### Flow cytometry

Expression of membrane Hsp70 on viable tumor cells with intact cell membranes was determined by flow cytometry following a protocol described elsewhere [18]. Briefly, after a washing step in flow cytometry buffer (PBS/10% v/v FBS), trypsinized single cells were incubated on ice with cmHsp70.1-FITC monoclonal antibody (mAb) (1:50, multimmune GmbH) for 30 min in the dark. After a further washing step cells were analyzed on a BD FACSCalibur™ instrument (BD Biosciences, Heidelberg, Germany). To exclude non-viable cells from analysis, propidium iodide (PI, 1 µg/mL, Sigma-Aldrich/Merck) was added directly before flow cytometric analysis. At least 2 × 10^4^ viable cells were aquired in each sample. Only viable (PI-negative) cells with an intact cell membrane were gated and analyzed. An IgG1 isotype matched FITC-labeled immunoglobulin (mouse IgG1-FITC, 345,815, BD Biosciences) was used to evaluate nonspecific binding. Membrane Hsp70 positivity was determined by subtracting the percentage of cells stained with the isotype-matched control antibody from that of the cells positively stained with the cmHsp70.1-FITC mAb.

### Irradiation

Tumor cells (LS174T, LoVo, A549, MDA-MB-231, Colo357) were irradiated with a single dose of 1, 2, 4 and 6 Gy using the Gulmay RS225A device (Gulmay Medical Ltd., Camberley, UK) at a dose rate of 1.1 Gy/min (15 mA, 200 kV) or were sham irradiated (0 Gy).

### Clonogenic cell survival assay

Tumor cells were seeded into 12-well plates, treated with 0.1 mM diclofenac for 48 h and irradiated with the indicated doses. After irradiation, the medium was removed and cells were cultured in fresh, drug-free medium. For analyzing the drug sensitivity tumor cells were treated with 0.1 mM diclofenac either alone or in combination with 1 or 5 µM 5-FU for 48 h. After 8–9 days, plates were washed in PBS, fixed with ice-cold methanol and colonies were stained using 0.1% w/v crystal violet. The number of colonies (≥ 50 cells) was counted automatically using a Bioreader® 3000 (Bio-Sys GmbH, Karben, Germany). Survival curves were fitted to the linear quadratic model using SigmaPlot (Systat Software Inc., San Jose, CA, USA).

### Murine xenograft tumor model and administration of diclofenac and irradiation

#### Animals and ethics statement

Female C57BL/6J mice (4–6 weeks old) were purchased from the Pasteur Institute of Iran. Animals had ad libitum access to food and water during maintenance under standard conditions (22 °C, 50% v/v relative humidity, and 12 h light/dark cycles). Mice were adapted to the standard housing conditions for one week before the start of the experiments. All animal procedures were performed in compliance with the revised Animals Directive 2010/63/EU of the European Union and were approved by the local ethical committee of the veterinary medicine faculty Shahid Chamran University of Ahvaz under the permit number EE/1401.2.24.97953/SCU.AC.IR.

#### Tumor cell injection

At a confluency of 70–80%, LS174T tumor cells were trypsinized using 0.5% v/v trypsin-EDTA for 5 min at 37 °C, followed by centrifugation at 400×g for 5 min. The supernatant was removed and the cells were washed twice with PBS. The cells were counted using a Neubauer chamber, and cell viability was determined by trypan blue exclusion. C57BL/6J mice were exposed to a standard whole-body irradiation with 3 Gy (Elekta compact, X-6 MV, Elekta Solutions AB, Stockholm, Sweden) to immunocompromise the animals and suppress residual immunity which enhances the engraftment and growth of xenograft tumor cells. After 24 h mice were anesthetized by an intraperitoneal injection of ketamine-xylazine 25.5 mg/mL (1 mL/kg body weight). LS174T tumor cells were suspended in 0.1 mL PBS (1 × 10^6^ cells/mice) and injected subcutaneously (s.c.) into the right shoulder region in a total of 24 mice. LS174T adenocolorectal cancer cells were chosen because a low concentration of diclofenac (0.1 mM) already showed a radiosensitizing effect and an inhibition of the HSF1, in vitro. The tumor volume at the site of injection was measured regularly every three days by a digital caliper using the formula of Volume (mm^3^) = (A) × (B^2^)/2, where A was the largest diameter (mm) and B the smallest (mm). Only mice whose tumors reached a size of 40 mm^3^ (*n* = 16, approximately 7 days after the tumor cell injection), were randomly divided into the following groups with 4 animals per group (*n* = 4): diclofenac, diclofenac-radiation, radiation and control group. The in vivo experiment was carried out once with a total of 16 mice. Eight of the 24 mice were euthanized and excluded from the experiment because the size of their tumors was too big. In the diclofenac and diclofenac-radiation groups, mice received three intraperitoneal injections of diclofenac (40 mg/kg) on days 7, 9 and 11. Then the tumors of the diclofenac-radiation and radiation animal groups were locally irradiated with 6 Gy. On day 16 all animals were euthanized by an intraperitoneal injection of sodium pentobarbital (800 mg/kg of body weight) because the tumors in the control group reached the maximum allowed tumor volume. Tumors and organs were excised on day 16 for further analysis.

### Statistics

Each experiment was repeated independently at least 3 times (biological replicates) if not otherwise indicated. The Student’s t-test was used to evaluate significant differences between two groups. One or two way ANOVA or Kruskal Wallis tests were used to evaluate significant differences between multiple groups (*p ≤ 0.05, **p ≤ 0.01, ***p ≤ 0.001). Data are presented as mean values with standard deviation (SD).

## Results

### The reduction in cell viability and c-MYC expression upon diclofenac treatment is concentration-dependent in different tumor cell lines

**Fig. 1:**
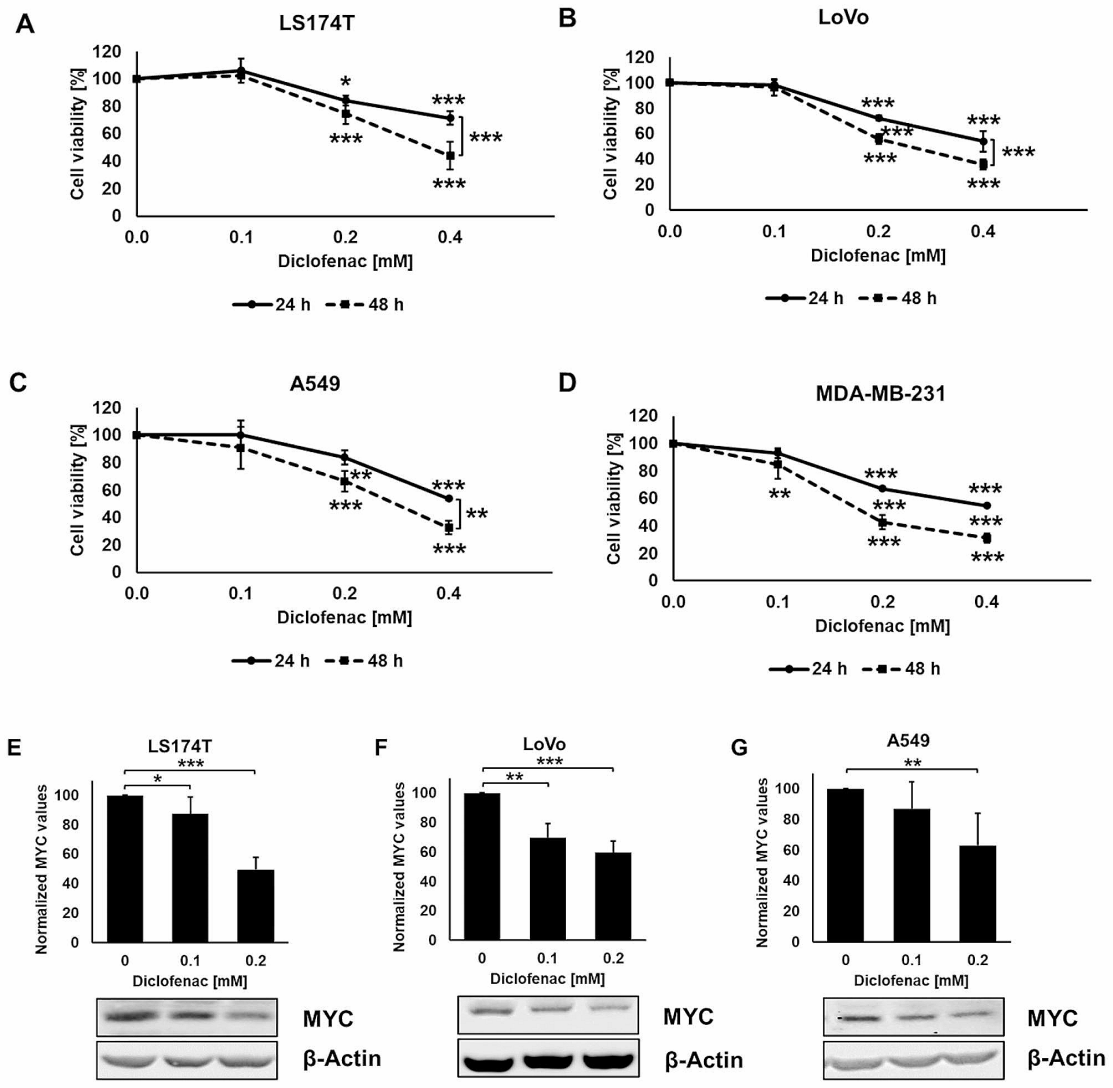
Diclofenac inhibits cell viability and c-MYC expression in LS174T, LoVo, A549 and MDA-MB-231 cancer cells. (**A-D**) Toxicity assay of LS174T (A), LoVo (B), A549 (C) and MDA-MB-231 (D) cancers cells treated with different diclofenac concentrations (0, 0.1, 0.2 and 0.4 mM) for 24 and 48 h. Two way ANOVA was used to evaluate significant differences (*p ≤ 0.05, **p ≤ 0.01, ***p ≤ 0.001). (**E-G**) Representative immunoblot showing the expression of c-MYC 48 h after diclofenac treatment in LS174T (E), LoVo (F) and A549 (G) cancer cells. Quantification of the c-MYC signals of at least 3 independent experiments are shown in the bar charts above (*p ≤ 0.05, **p ≤ 0.01, ***p ≤ 0.001)

NSAIDs exert anti-tumor effects which are mainly attributed to the inhibition of COX1/2 [1, 27, 28]. To study COX1/2 independent effects of diclofenac, two colorectal adenocarcinoma cell lines (LS174T and LoVo), the A549 lung cancer cell line and the MDA-MB-231 breast cancer cell line were cultured for 24 and 48 h with clinically relevant concentrations of diclofenac (0.1–0.4 mM) (http://www.drugs.com/pro/diclofenac.html) [4]. A concentration of 0.1 mM diclofenac did not significantly affect cell viability, whereas concentrations of 0.2 and 0.4 mM impaired cell viability in vitro in all four tumor cell types (Fig. 1A-D). In the COLO357 pancreatic cell line the low concentration of 0.1 mM diclofenac already caused a significant loss in cell viability (Supplementary Fig. 1). According to the findings of other groups, c-MYC expression is inhibited in melanoma, lymphoma and prostate carcinoma cells by diclofenac at concentrations in the range of 0.2 mM to 0.4 mM [4]. We could show that in the LS174T and LoVo colorectal adenocarcinoma cell lines c-MYC expression was already significantly reduced at the non-lethal concentration of 0.1 mM of diclofenac (Fig. 1E, F), but not in A549 cells. In A549 cells a concentration of 0.2 mM was necessary to reduce c-MYC (Fig. 1G).

### A non-lethal concentration of diclofenac inhibits LDH activity in colorectal adenocarcinoma cell lines, but not in lung, breast and pancreatic cancer cells

**Fig. 2:**
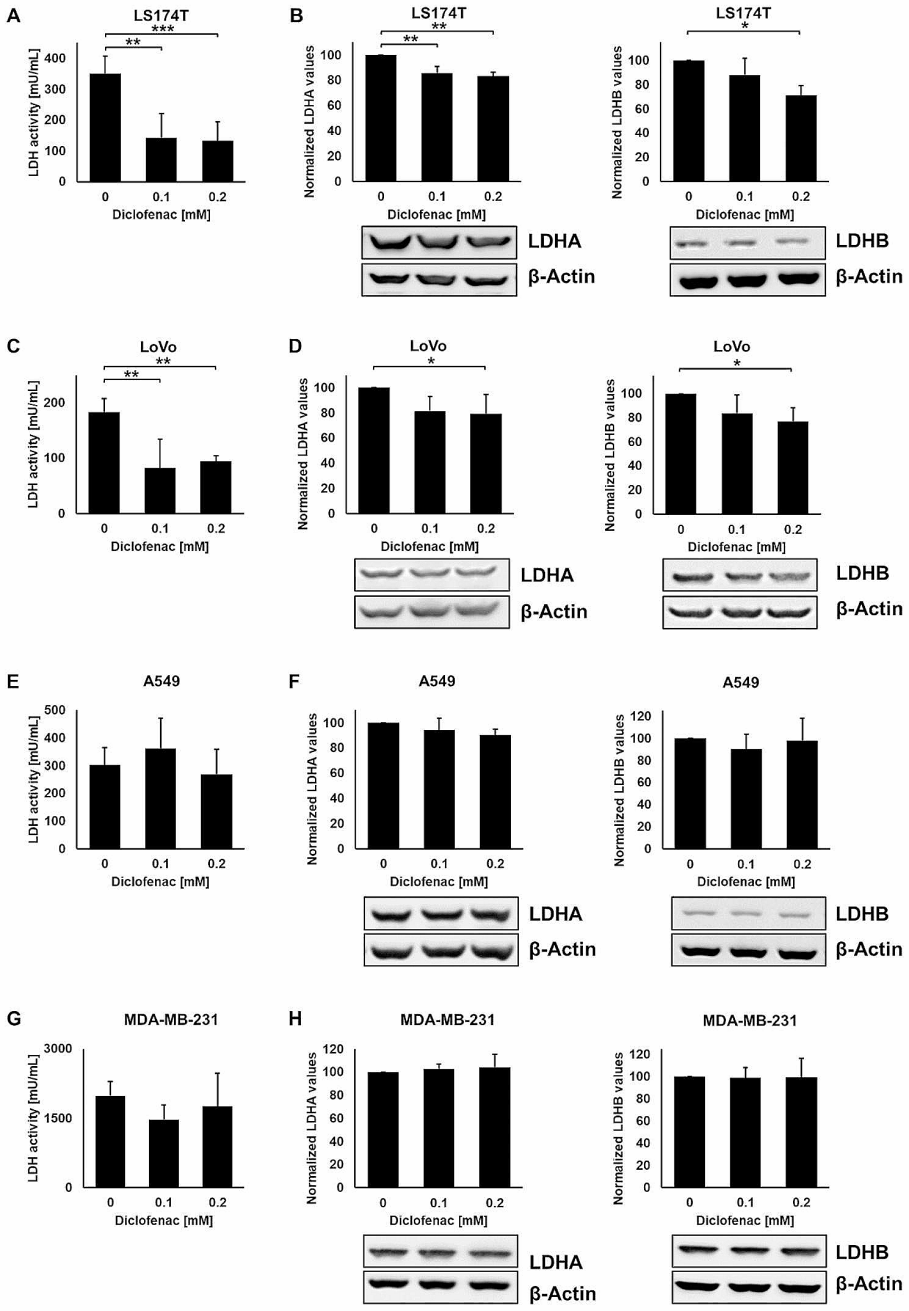
LDH activity and expression levels in LS174T, LoVo, A549 and MDA-MB-231 cancer cells after diclofenac treatment. LDH activity assay of LS174T (**A**), LoVo (**C**), A549 (**E**) and MDA-MB-231 (**G**) cancer cells treated with diclofenac (0, 0.1 and 0.2 mM) for 48 h. Representative immunoblot showing the expression of LDHA and LDHB 48 h after diclofenac treatment in LS174T (**B**), LoVo (**D**), A549 (**F**) and MDA-MB231 (**H**) cancer cells. Quantification of the LDHA and LDHB signals of at least 3 independent experiments are shown in the bar charts above. The one way ANOVA test was used to evaluate significant differences (*p ≤ 0.05, **p ≤ 0.01, ***p ≤ 0.001)

Since MYC regulates the synthesis of glycolytic enzymes including LDHA and thereby promotes aerobic glycolysis [29], LDH activity and LDHA/B protein expression were analyzed after diclofenac treatment. After a treatment with 0.1 and 0.2 mM diclofenac LDH activity and LDHA/B protein levels were significantly reduced in LS174T and LoVo colorectal cancer cells (Fig. 2A-D), but remained unaltered in A549 lung cancer (Fig. 2E, F), MDA-MB-231 breast cancer (Fig. 2G, H) and COLO357 pancreatic cancer cells (Supplementary Fig. 2A, B).

### Diclofenac-induced LDH inhibition is associated with an impaired stress response in colorectal adenocarcinoma cells, but not in lung, breast and pancreatic cancer cells

**Fig. 3:**
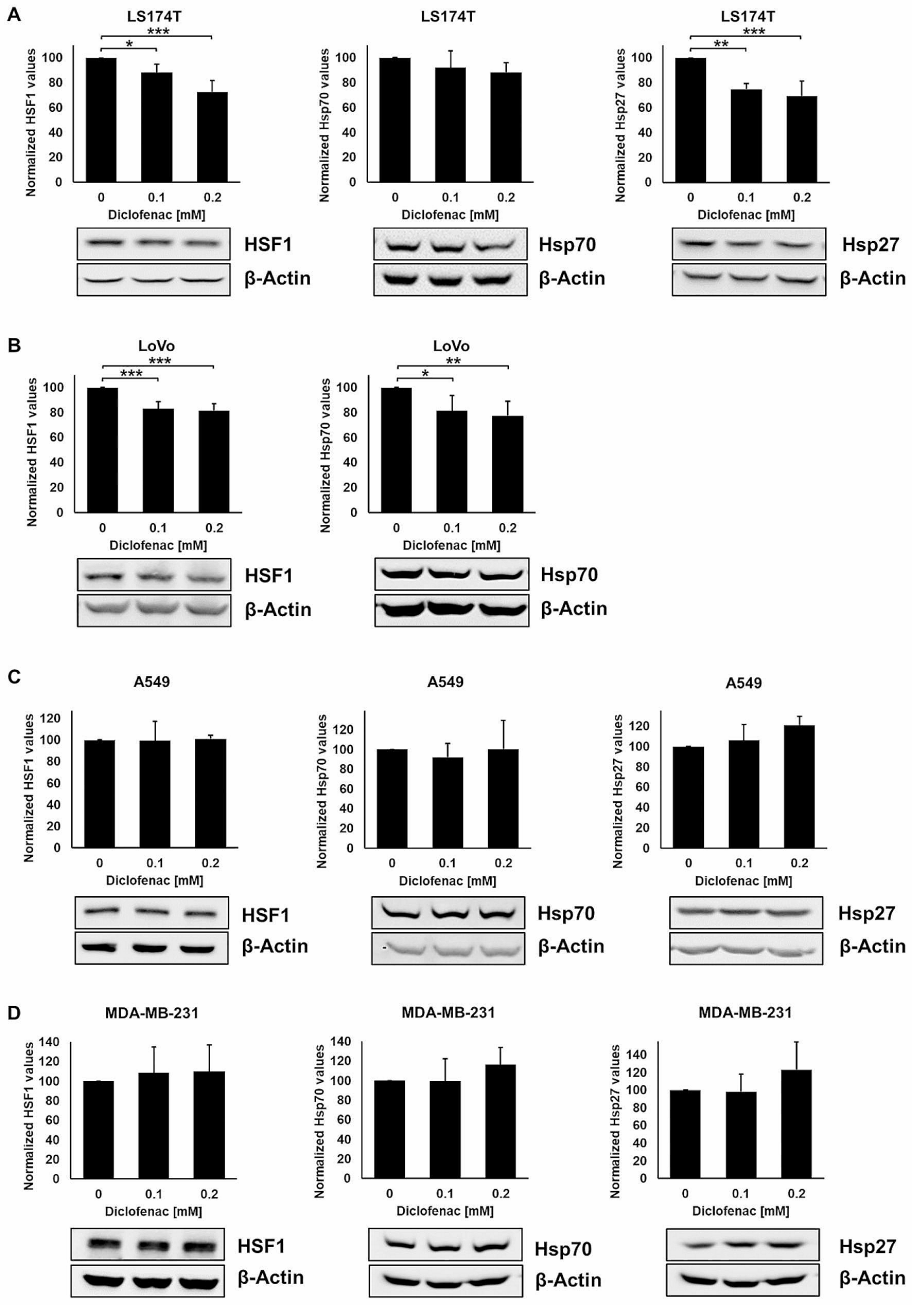
Effects of diclofenac on the cytosolic stress protein expression. Representative immunoblot showing the expression of HSF1, Hsp70 and Hsp27, 48 h after diclofenac treatment in LS174T (**A**), LoVo (**B**), A549 (**C**) and MDA-MB-231 (**D**) cancer cells. Quantification of the respective protein signals of at least 3 independent experiments are shown in the bar charts above. The one way ANOVA test was used to evaluate significant differences (*p ≤ 0.05, **p ≤ 0.01, ***p ≤ 0.001)

Since an inhibited lactate metabolism correlates with a reduced heat shock response [18] the expression of the transcription factor HSF1 and the major stress proteins Hsp70 and Hsp27 were analysed after treatment with a non-lethal concentration of diclofenac. LDH activity and the expression of HSF1, Hsp70 and Hsp27 decreased in the LS174T and LoVo colorectal adenocarcinoma cell lines (Figs. 2A-D and 3A and B) with lower basal HSP levels, but not in A549 (Figs. 2E-F and 3C), MDA-MB-231 (Figs. 2G-H and 3D) and COLO357 cells (Supplementary Fig. 2A, B, Supplementary Fig. 3). Due to very low basal levels of Hsp27 in LoVo cells a quantification of this stress protein was not possible by Western blot analysis.

### Diclofenac-mediated LDH inhibition is associated with a reduction in the Hsp70 membrane positivity in colorectal adenocarcinoma cells, but not in lung, breast and pancreatic cancer cells

**Fig. 4:**
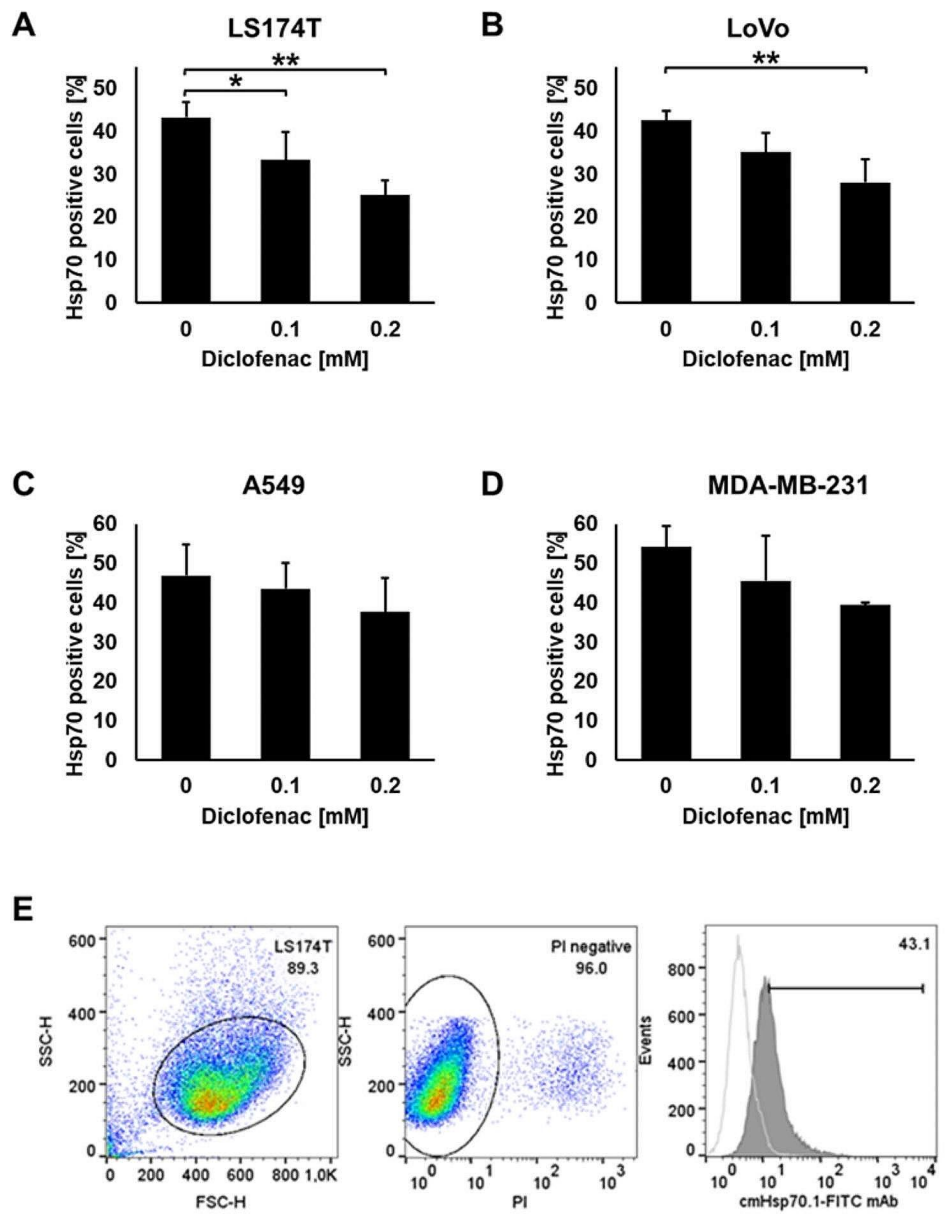
Effects of diclofenac on membrane Hsp70 expression. Plasma membrane Hsp70 expression on untreated and diclofenac treated (0.1, 0.2 mM for 48 h) LS174T (**A**), LoVo (**B**), A549 (**C**) and MDA-MB-231 (**D**) cancer cells. Data present the proportion of positively stained cells of at least 3 independent experiments. (**E**) Representative gating strategy to determine membrane Hsp70 expression on viable tumor cells. Left, side scatter (SSC)/forward scatter (FSC) dot plot histogram to identify the tumor cell population based on size (FSC) and granularity (SSC); middle, gating of Propidium Iodide (PI) negative, viable tumor cells (96.0%); right, overlay of two histograms representing membrane Hsp70 positive tumor cells (43.1%) using cmHsp70.1-FITC monoclonal antibody (mAb, gray) and the negative control (white histogram) using an isotype-matched mAb (anti-mouse IgG1-FITC). The one way ANOVA test was used to evaluate significant differences (*p ≤ 0.05, **p ≤ 0.01)

Due to a tumor-specific lipid composition [30] tumor cells, but not normal cells, present Hsp70 on their plasma membrane [31, 32]. Concomitant with a significant decrease in the cytosolic expression of the major stress protein Hsp70, LS174T and LoVo cells also showed a significantly decreased membrane Hsp70 positivity after diclofenac treatment (Fig. 4A, B). In contrast, the cytosolic as well as membrane Hsp70 levels in A549 (Fig. 4C), MDA-MB-231 (Fig. 4D) and COLO357 cells (Supplementary Fig. 4) remained unaltered. A representative example of a gating strategy to determine membrane Hsp70 expression on viable LS174T tumor cells is shown in Fig. 4E.

### Diclofenac-mediated inhibition of LDH significantly increases radio- and chemo-sensitivity in colorectal adenocarcinoma cells

**Fig. 5:**
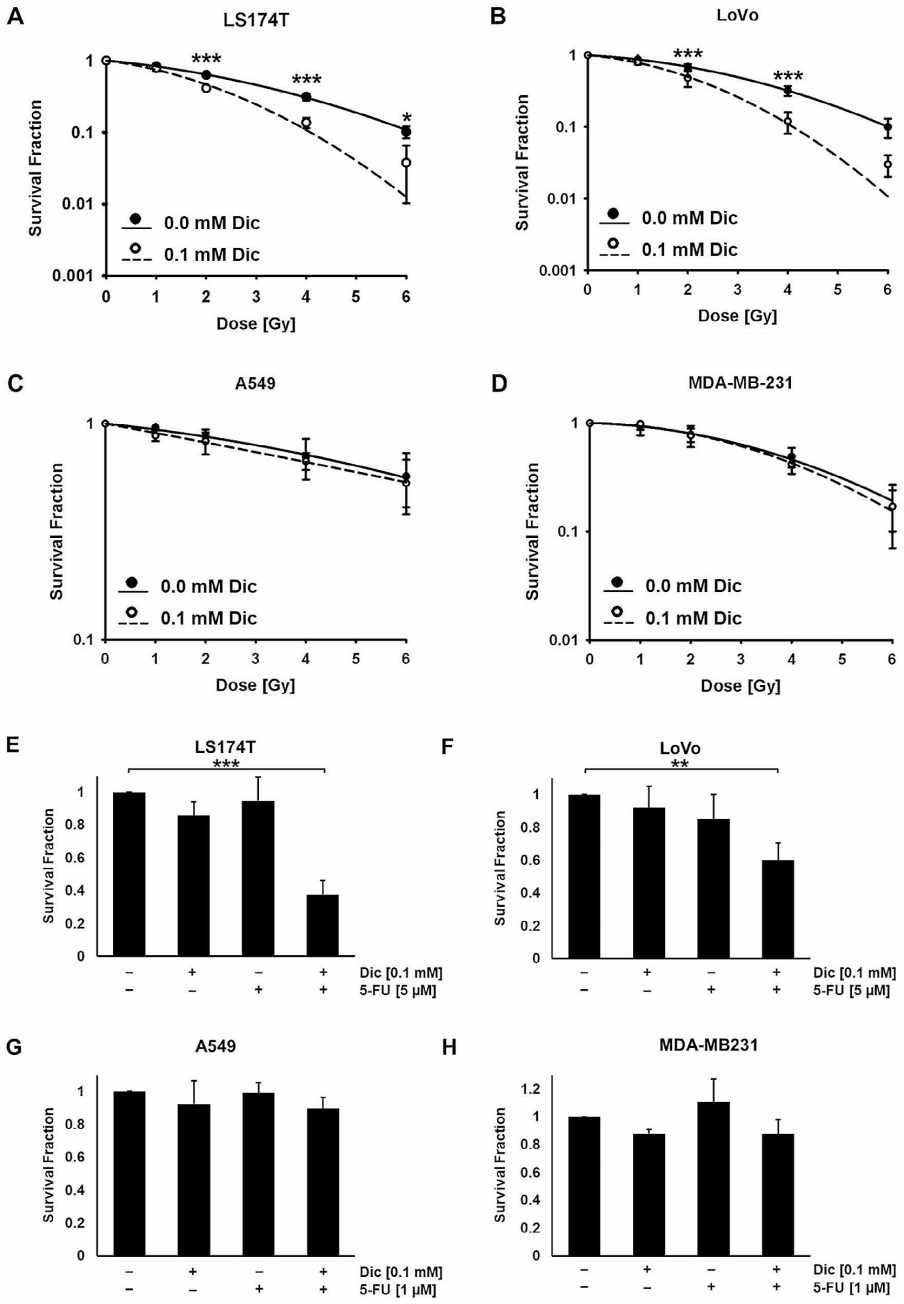
Colony forming assay for LS174T, LoVo, A549 and MDA-MB-231 cells. LS174T (**A**), LoVo (**B**), A549 (**C**) and MDA-MB-231 (**D**) cancer cells were treated with 0.1 mM diclofenac (Dic) for 48 h and then were irradiated (0–6 Gy). The cells were allowed to form colonies in drug-free medium. Two way ANOVA was used to evaluate significant differences (*p ≤ 0.05***, *p* ≤ 0.001). LS174T (**E**), LoVo (**F**), A549 (**G**) and MDA-MB-231 (**H**) were kept untreated or were treated with 0.1 mM diclofenac, 1 or 5 µM 5-fluorouracil (5-FU) or with diclofenac and 5-FU. After 48 h the medium was changed and cells were allowed to form colonies. The colony forming assay represents the results of at least 3 independent experiments. The one way ANOVA test was used to evaluate significant differences (**p ≤ 0.01, ***p ≤ 0.001)

Radioresistance of cancer cells is not only mediated by increased cytosolic Hsp70 levels, but also by an increased plasma membrane expression of Hsp70 [20, 33], with membrane Hsp70 having been shown to contribute to the membrane stability upon stress [34]. In this study we could show that diclofenac, even at low concentrations, inhibits both the cytosolic and membrane expression of Hsp70 in colorectal adenocarcinoma cells. Therefore, we investigated the radio-sensitizing effects of diclofenac. After an incubation of LS174T, LoVo, A549, MDA-MB-231 and COLO357 cells with 0.1 mM diclofenac for 48 h, cells were irradiated (0, 2, 4, 6 Gy). As shown in Fig. 5A and B, a non-lethal concentration of diclofenac significantly sensitizes LS174T and LoVo cells towards radiation in a clonogenic cell survival assay (Fig. 5A, B). These findings are associated with a significant reduction in the D_50_ value and a sensitizing enhancement ratio (SER) of more than 1.20 (Supplementary Table 1). In contrast to the colorectal cancer cells, this radio-sensitizing effect was not observed in A549 lung cancer (Fig. 5C, Supplementary Table 1), MDA-MB-231 breast cancer (Fig. 5D, Supplementary Table 1) and COLO357 pancreatic cancer cells (Supplementary Fig. 5) which also show no changes in the lactate and stress response upon diclofenac treatment. Moreover, radiation alone (2 Gy, 3 Gy) did not impact the LDH levels in LS174T and A549 cells (data not shown).

A comparison of the production of reactive oxygen species (ROS) revealed that a low concentration of diclofenac or irradiation alone did not significantly alter ROS levels, whereas a combined treatment with diclofenac (0.1 mM) and irradiation with 2 and 3 Gy, respectively, significantly increased the ROS production in colorectal adenocarcinoma cells (LS174T) already at 2 Gy, but not in lung carcinoma cells (A549) even at 3 Gy (Supplementary Fig. 6).

Since metastatic colorectal cancers are commonly treated with the chemotherapeutic agent 5-FU [35], we also investigated the effects of diclofenac on drug sensitivity. Since A549 and MDA-MB-231 cells are more sensitive to 5-FU than the colorectal adenocarcinoma cells, they were treated with a lower concentration of 5-FU [1 µM] than the LS174T and LoVo cells [5 µM]. Colony forming assays revealed that 0.1 mM diclofenac sensitizes LS174T (Fig. 5E) and LoVo (Fig. 5F) colorectal cancer cells against 5FU, but not A549 lung cancer cells (Fig. 5G) and MDA-MB-231 breast cancer cells (Fig. 5H). These results suggest that diclofenac not only induces radiation, but also chemo-sensitization in colorectal cancer cells. The chemo-sensitizing effect of diclofenac in colorectal cancer cells was associated with an impaired LDH activity and stress response.

Diclofenac enhances the effect of radiotherapy in a murine xenograft tumor model.

**Fig. 6:**
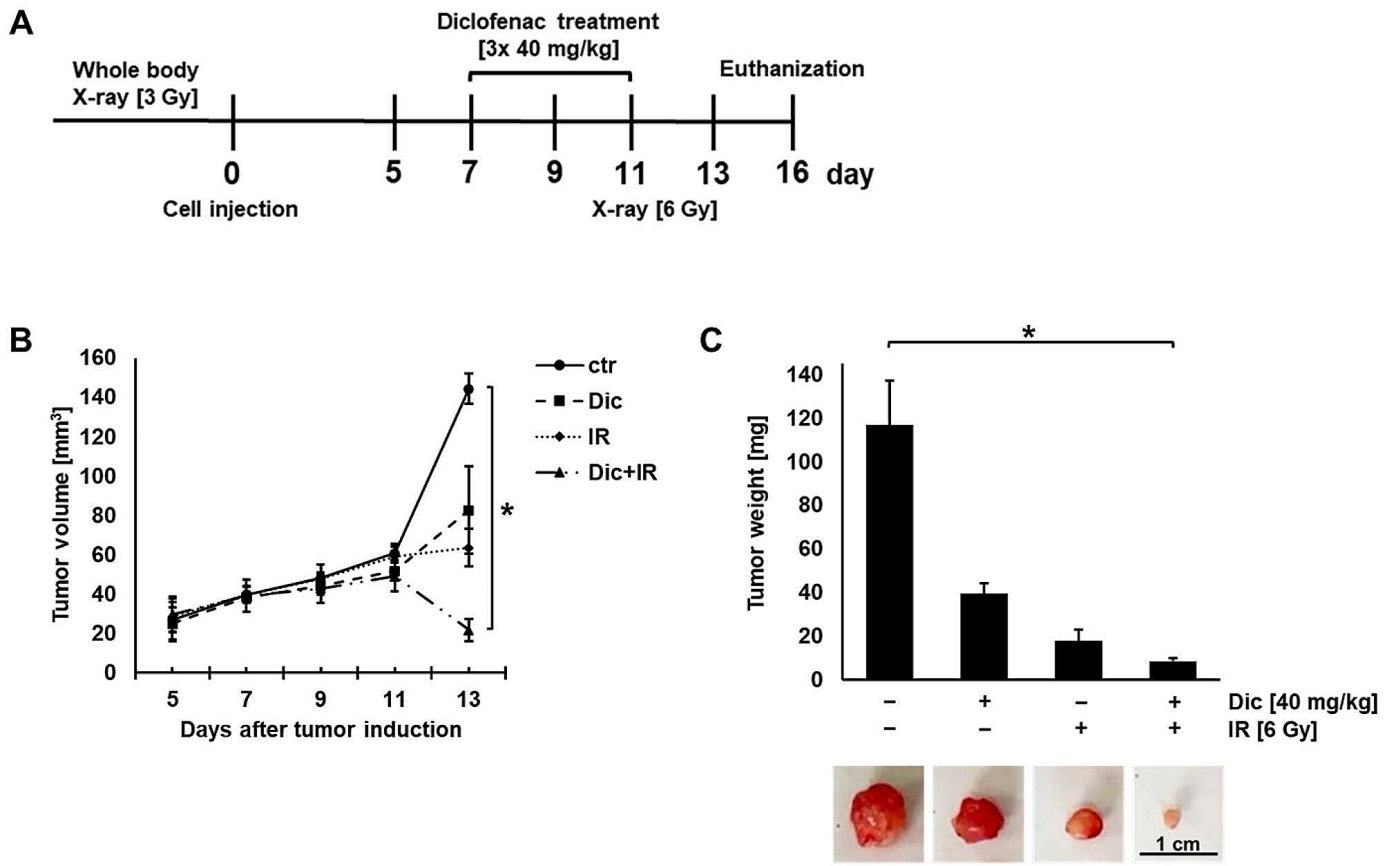
Diclofenac enhances the effect of radiotherapy in a LS174T xenograft mouse model. (**A**) Treatment schedule. C57/BL6J mice were exposed to a whole-body irradiation (3 Gy) to immunocompromise the animals. 24 h after irradiation, LS174T cells were injected subcutaneously (s.c.) into the right shoulder region. On day 7 after the tumor cell injection mice with identical tumor sizes were randomly divided into the following four groups each with 4 animals: control (ctrl, *n* = 4), diclofenac (Dic, *n* = 4), radiation (IR, *n* = 4) and diclofenac-radiation (Dic + IR, *n* = 4). In the diclofenac and diclofenac-radiation groups, mice received intraperitoneal injections of diclofenac (40 mg/kg) on days 7, 9 and 11. Then the diclofenac-radiation and radiation groups were locally irradiated with a single dose of 6 Gy on day 11. On day 16 all animals were euthanized and the tumors and organs were excised for analysis. (**B**) Tumor volume was measured regularly every 3 days with a digital caliper. Kruskal Wallis Test was used to evaluate significant differences (*p ≤ 0.05). (**C**) After euthanization the weights of the mice and the excised tumors were determined. Kruskal Wallis Test was used to evaluate significant differences (*p ≤ 0.05)

To confirm the radio-sensitizing effect of diclofenac in vivo, immunocompromised mice bearing subcutaneous LS174T tumors were injected on days 7, 9 and 11 intraperitoneally with diclofenac (40 mg/kg). On day 11 tumors were irradiated locally with 6 Gy (Fig. 6A). The tumor size was measured regularly every three days by caliper measurements. As shown in Fig. 6B, the fastest tumor growth was observed in the control group. A treatment with diclofenac or irradiation alone resulted in a decreased tumor size, but the best tumor control was achieved in mice who received both, diclofenac and irradiation (Fig. 6B). As shown in Fig. 6C, on day 16 the tumor weight and size were significantly reduced in the group of mice who received the combined treatment consisting of diclofenac and irradiation. A histopathological inspection of the organs of the mice (intestine, stomach, kidney, lung, and liver) treated with diclofenac revealed no pathological lesions after treatment with diclofenac (Supplementary Fig. 7).

## Discussion

The metabolic reprogramming of many cancer cells is associated with an increased glucose uptake and an elevated aerobic glycolysis known as the “Warburg effect” leading to high lactate levels and acidosis in the TME [5, 8]. Activation of the PI3K-Akt-mTORC1 signalling pathway, loss of p53 and an overexpression of Hypoxia-Inducible Factor 1 (HIF1) and c-MYC facilitate these biochemical alterations [36]. HIF1 and c-MYC favour the glycolytic pathway by promoting the expression of target genes like *LDHA* [8, 37]. Although in a murine A549 lung cancer xenograft model, a genetic (*LDHA* knockout) and chemical (tamoxifen) inactivation of LDHA has been shown to inhibit tumorigenesis [38], very low concentrations of diclofenac (0.1, 0.2 mM) had no effect on LDHA/B expression or the stress response (HSF-1, Hsp70, Hsp27) in lung (A549), breast (MDA-MB-231) and pancreatic (Colo357) cancer cells, but in adenocolorectal (LS174T, LoVo) cancer cells. This finding is due to the fact that a low concentration of diclofenac (0.1 mM) in combination with a low irradiation dose significantly increased the ROS production in adenocolorectal (2 Gy; LS174T), but not in lung (3 Gy; A549) cancer cells (supplementary Fig. 6). In addition, the basal levels of anti-apoptotic stress proteins such as Hsp27 and Hsp70, both regulated by HSF1, are lower in colorectal cancers than in lung and breast cancer cells.

Despite significant efforts to identify inhibitors of key glycolytic enzymes and promising preclinical data, only a limited amount of drugs which have the potential to break radio- and chemo-resistance and lactate-mediated immunosuppression are presently used in clinical practise [39, 40]. Previous studies have indicated that the clinically approved NSAID diclofenac can inhibit glycolytic genes such as the *Glucose Transporter 1* (*GLUT1*), *LDHA* and *Monocarboxylate Transporter 1* (*MCT1*) [4], and thereby reduce the uptake of glucose and the production of the oncometabolite lactate [2, 4]. The inhibition of glycolytic genes in colorectal cancer cells by diclofenac might be explained by a reduction in c-MYC expression that alters STAT-3 signalling through its decreased phosphorylation. MYC, a downstream target of STAT-3, is a regulator of glycolytic enzymes including LDHA. Furthermore, diclofenac alters the lactate efflux and leads to an intracellular accumulation of lactate by inhibiting the function of MCT1 due to its monocarboxylic acid structure [4, 41]. We have shown that a low concentration of dioclofenac (0.1 mM) negatively affected the c-MYC expression in LS174T and LoVo colorectal cancer cells (Fig. 1E-F), but not in A549 lung cancer cells. Based on these findings an LDH inhibition concomitant with a reduction in anti-apoptotic stress proteins by low concentrations of diclofenac was observed in colorectal (LS174T, LoVo, Fig. 2A, C) cancer cells, but not in lung (A549, Fig. 2E), breast (MDA-MB-231, Fig. 2G) and pancreatic (Colo357, Supplementary Fig. 2) cancer cells. An association of an impaired LDH activity and a reduced stress response has been confirmed in tumor cells with a genetic knockout of *LDHA* and *LDHB* [18].

As early as 1994, Hixsen and colleagues showed that NSAIDs including diclofenac exert anti-proliferative activity on human colon cancer cell lines [42]. Our study confirmed that above a concentration of 0.1 mM, diclofenac induces cytotoxic effects in LS174T, LoVo, A549 and MDA-MB-231 cells (Fig. 1A-D). To avoid adverse effects (i.e. gastrointestinal complications) the radio- and chemo-sensitizing potential of diclofenac was studied at the low, non-lethal concentration of 0.1 mM.

A link between the glucose/lactate metabolism and the stress response has previously been shown by different groups [18, 43, 44]. Herein, we demonstrate that a non-lethal concentration of diclofenac reduces the cytosolic expression of HSF1, Hsp70 and Hsp27 and the plasma membrane expression of Hsp70 in LS174T and LoVo cells (Figs. 3A and B and 4A and B), but not in A549 (Figs. 3C and 4C), MDAMB-231 (Figs. 3D and 4D) and COLO357 cells (Supplementary Fig. 3, Supplementary Fig. 4). The stress response is an important survival mechanism to protect tumor cells from death induced by physical or chemical stress factors such as heat, radiation and oxygen radicals [17]. Many tumor cells overexpress stress proteins in the cytosol to protect them against lethal damage induced by environmental stress interfering with apoptotic pathways [16] and by stabilizing DNA repair proteins [45]. Therefore, impairing the cellular stress response is considered as a promising strategy to break radio- and/or chemo-resistance of tumor cells by enabling apoptosis and inhibiting DNA repair. In recent years, different inhibitors of Hsp90, Hsp70 and Hsp27 have been investigated in preclinical and clinical studies with mixed responses [46]. Our laboratory recently showed that the Hsp90 inhibitor NVP-AUY922 potentiate the radiosensitivity in cancer cells with an impaired lactate metabolism [47]. Due to the redundancy of the HSP network, an inhibition of Hsp90 results in an upregulated expression of the anti-apoptotic chaperone Hsp70 [48]. Moreover, most of the Hsp90 inhibitors are not soluble in aqueous solutions and induce hepatotoxicity [49]. Therefore, the clinical approved NSAID diclofenac at very low concentrations might serve as an attractive candidate for addressing both, pro-tumorigenic (lactate metabolism) and anti-apoptotic (stress protein synthesis) mechanisms. An overexpression of stress proteins in the cytosol and on the cell surface of tumor cells contributes to therapy resistance [16, 33]. We demonstrated that diclofenac at non-lethal concentrations not only reduces the cytosolic and membrane Hsp70 expression, but also contributes to radio- and chemo-sensitization in colon cancer cells.

Studies demonstrated that NSAIDs exert radio-sensitizing activities by stimulating reoxygenation within the tumor [50]. Furthermore, topical application of diclofenac was shown to exert radio-sensitizing effects in vitro and in vivo in COX2 overexpressing prostate cancer cells [51]. However, at low concentrations (0.1–0.2 mM), diclofenac uncouples the mitochondrial energy metabolism, most likely via COX-dependent pathways [52].

In normal cells, NSAIDs including diclofenac can also mediate radioprotective, anti-oxidative effects that reduce radiation-induced toxicity [53]. This ability is based on the fact that diclofenac has at least an additive antioxidant free-radical scavenging activity as proven for human erythrocytes [54] and serum albumin [55]. Diclofenac scavenges radiation-induced oxidative stress and inflammation in normal tissues, particularly in the vascular system [53–55]. Therefore, diclofenac might have dual functions, on the one hand it prevents vascular inflammation and on the other side it radiosensitizes tumor cells.

In addition to its radiosensitizing effects, NSAIDs have been shown to increase chemosensitivity in colorectal adenocarcinoma cells through an upregulation of the pro-apoptotic protein BAX [56]. In an in vitro TK6 cell-based assay, diclofenac has been shown to induce DNA double strand breaks as determined by an increase in γH2AX positive cells, micronuclei and a nuclear translocation of p53 [57] which might indicate that diclofenac acts synergistically with the DNA damage response caused by radiotherapy.

In our study, a non-lethal concentration of diclofenac was found to increase the radio- and chemo-sensitivity in two colorectal adenocarcinoma cell lines LS174T and LoVo (Fig. 5A, B, E-F). In contrast, in A549 lung carcinoma cells (Fig. 5C, G), MDA-MB-231 breast cancer cells (Fig. 5D, H) and COLO357 pancreatic cancer cells (Supplementary Fig. 5) diclofenac showed no radio- and chemo-sensitizing potential. Radiation alone (2 to 6 Gy) has not been shown to alter the cytosolic expression of Hsp70, the major stress-inducible member of the 70 kDa HSP family, in several tumor entities including glioblastoma, cervival cancer hepatocellular carcinoma, colon and lung cancer [58, 59], as well as the LDH activity.

The radio- and chemo-sensitizing effect in colorectal cancer cells could be attributed to an interference of diclofenac with the aerobic glycolysis, via an inhibition of LDH activity, to lower basal levels of anti-apoptotic stress proteins than lung, breast and pancreatic cancer cells and to an increased ROS production after a combined treatment with a low concentration of diclofenac (0.1 mM) and a low irradiation dose (2 Gy).

In line with the in vitro data, a diclofenac treatment also radiosensitizes LS174T colorectal adenocarcinomas with a reduced LDH activity and HSF1 expression in a murine xenograft tumor model (Fig. 6B, C). The tumor cell line LS174T was used in the xenograft tumor mouse model because a radiosensitizing effect by diclofenac could be demonstrated for this cell line in vitro (Fig. 5), and because a *LDHA* and *LDHB* gene knockout resulted in an increased radiosensitivity [18]. Previous results derived from a LS174T tumor mouse model indicated that a radiation dose of 5 × 7 Gy results in complete tumor control [60]. In order to get an adequate therapeutic window, mice with LS174T tumors were irradiated with a much lower dose. The single irradiation dose of 6 Gy for the in vivo irradiation of LS174T tumors is based on the D_50_ value of 2.43 Gy in LS174T cells, in vitro [18] and because the inhibitory effect on tumor growth by irradiation and a repeated treatment with diclofenac was comparable (as demonstrated in a pre-study). This finding enabled us to compare singular and combined effects induced by irradiation and a diclofenac treatment.

## Conclusion

NSAIDs are clinically approved and widely used for the treatment of pain and inflammatory conditions. We show that a well-tolerated, non-toxic concentration of diclofenac harbours the potential of breaking radio- as well as chemo-resistance in tumor types in which diclofenac decreases the LDH activity and stress response. Therefore, we propose that monitoring LDH activity and stress response might predict the radio- and chemo-sensitizing potential of diclofenac.

### Electronic supplementary material

Below is the link to the electronic supplementary material.

Supplementary Material 1

Supplementary Material 2

## Data Availability

All data generated or analyzed during this study are included in this published article and its supplementary information files.

## Abbreviations

5-FU: 5-Fluorouracil
CCK8: Cell Counting Kit8
COX: Cyclooxygenase
DMEM: Dulbecco`s Eagle`s Minimum Essential Medium
FBS: Fetal Bovine Serum
GLUT1: Glucose Transporter 1
HIF1: Hypoxia-inducible Factor 1
HSF1: Heat Shock Factor 1
HSP: Heat Shock Protein (family)
LDH: Lactate Dehydrogenase
LDHA: Lactate Dehydrogenase A
LDHB: Lactate Dehydrogenase B
MCT1: Monocarboxylate Transporter 1
NSAID: Nonsteroidal anti-inflammatory drug
PBS: Phosphate Buffered Saline
RPMI: Roswell Park Memorial Institute
TME: Tumor microenvironment
