# Supplementary material for: The radiation- and chemo-sensitizing capacity of diclofenac can be predicted by a decreased lactate metabolism and stress response
Source: Radiat Oncol. 2024 Jan 16;19:7. doi: 10.1186/s13014-024-02399-5 (PMC10790495; doi:10.1186/s13014-024-02399-5)
Supplement: Supplementary file 1 — Supplementary Material 1 [file 13014_2024_2399_MOESM1_ESM.docx]

Supplementary Figures and Tables


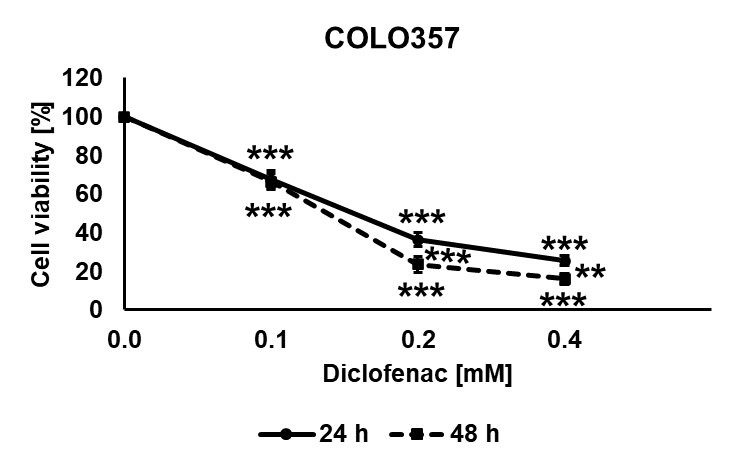


**Supplementary Figure 1.** Diclofenac inhibits cell viability in COLO357 cancer cells. Toxicity assay COLO357 cancers cells treated with diclofenac (0.0, 0.1, 0.2 and 0.4 mM) for 24 and 48 h. Two way ANOVA was used to evaluate significant differences (**p ≤ 0.01, ***p ≤ 0.001).


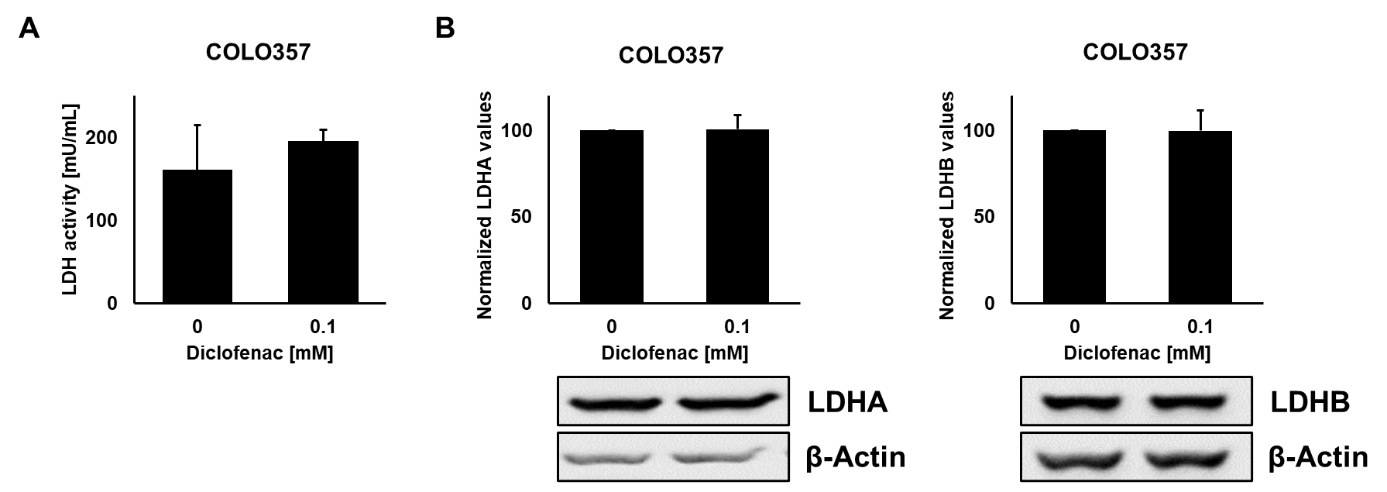


**Supplementary Figure 2.** LDH activity and expression levels in COLO357 cancer cells after diclofenac treatment. (A) LDH activity assay of COLO357 cancer cells treated with diclofenac (0 and 0.1 mM) for 48 h. (B) Representative immunoblot showing the expression of LDHA and LDHB 48 h after diclofenac (0 and 0.1 mM) treatment. Quantification of the LDHA and LDHB signals of at least 3 independent experiments are shown in the bar charts above.


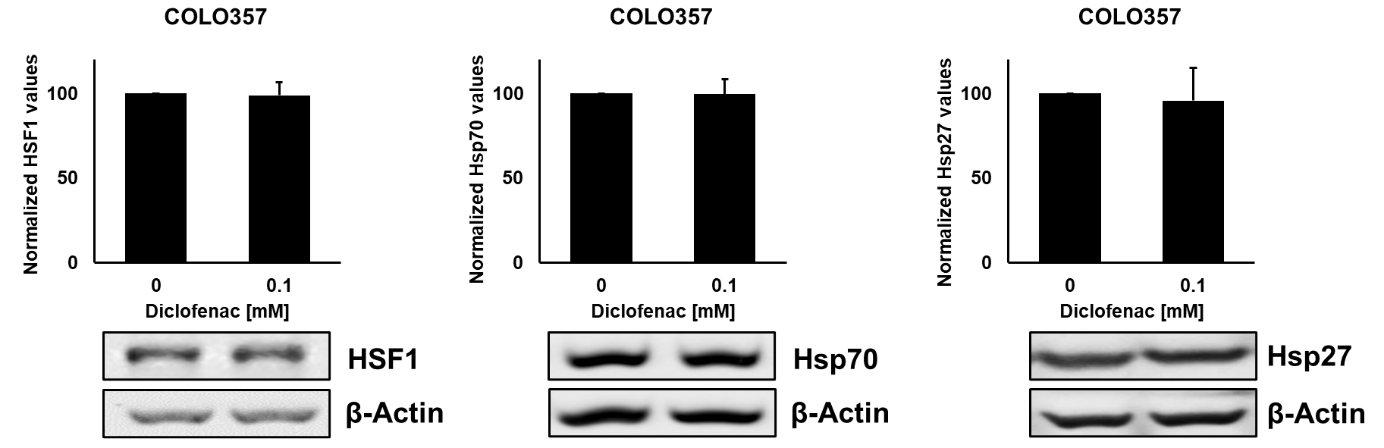


**Supplementary Figure 3.** Effects of diclofenac on the cytosolic stress protein expression. Representative immunoblots showing the expression of HSF1, Hsp70 and Hsp27 48 h after diclofenac (0 and 0.1 mM) treatment in COLO357 cancer cells. Quantification of the representative protein signals of at least 3 independent experiments are shown in the bar graphs above.


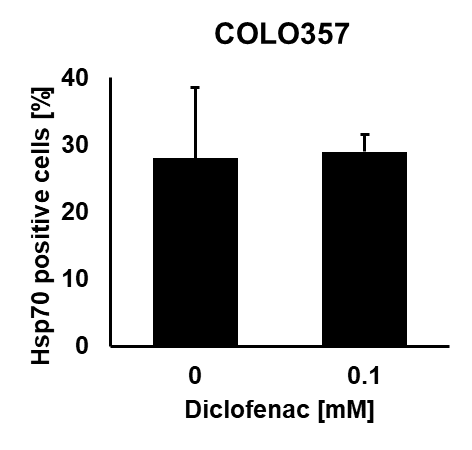


**Supplementary Figure 4.** Effects of diclofenac on the membrane Hsp70 expression. Plasma membrane bound Hsp70 on untreated (0 mM) and diclofenac treated (0.1 mM for 48 h) COLO357 cancer cells. The proportion of positively stained cells is shown.


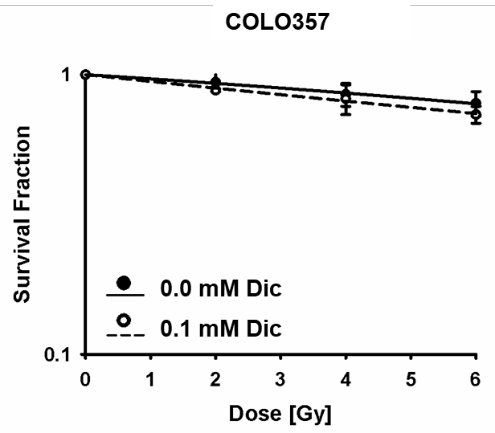


**Supplementary Figure 5.** Colony forming assay for COLO357 cancer cells. Cells were treated with 0.1 mM diclofenac (Dic) for 48 h, irradiated (0-6 Gy) and then allowed to form colonies in drug-free medium. Data represent mean values of 3 independent experiments.


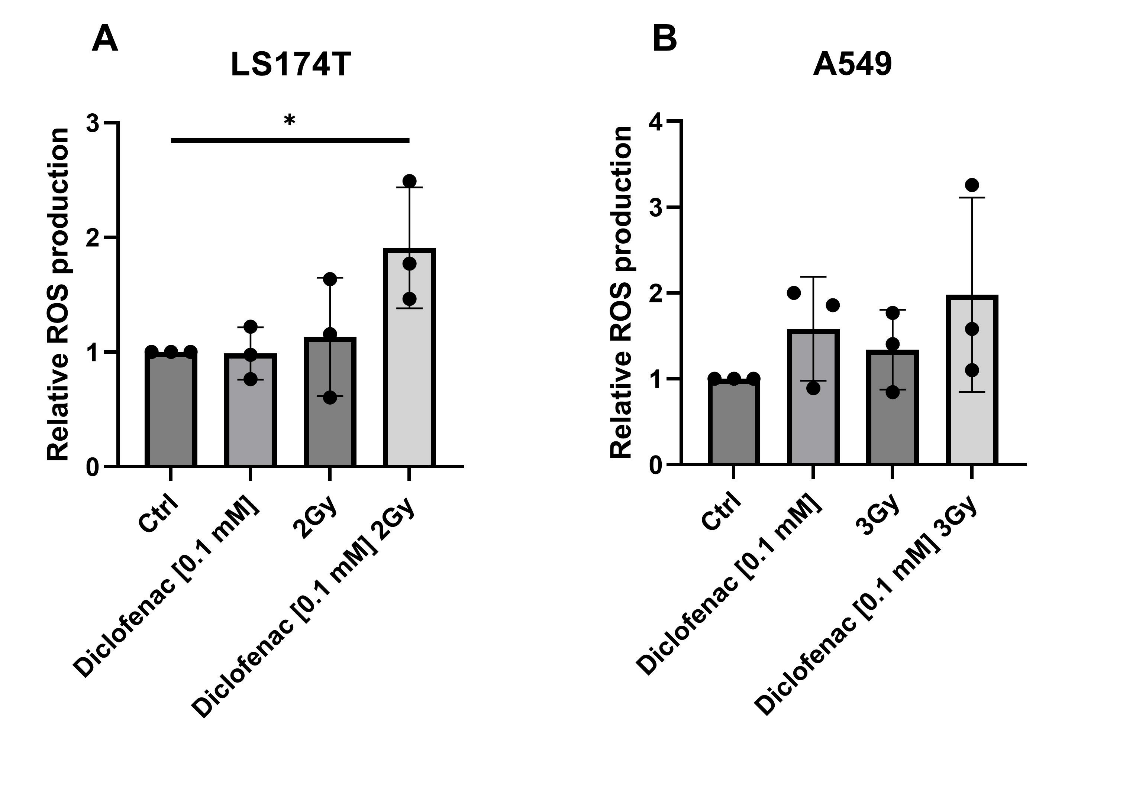


**Supplementary Figure 6.** Effects of diclofenac (0.1 mM for 48 h), radiation (2, 3 Gy) and a combined treatment of diclofenac followed by irradiation on the ROS production of LS174T (A) and A549 cells (B) as determined by the mean fluorescence intensity using the DCFDA kit. Data show the relative ROS production of 3 experiments. One way ANOVA was used to evaluate significant differences between control and the different treatments (*p ≤ 0.05).

**
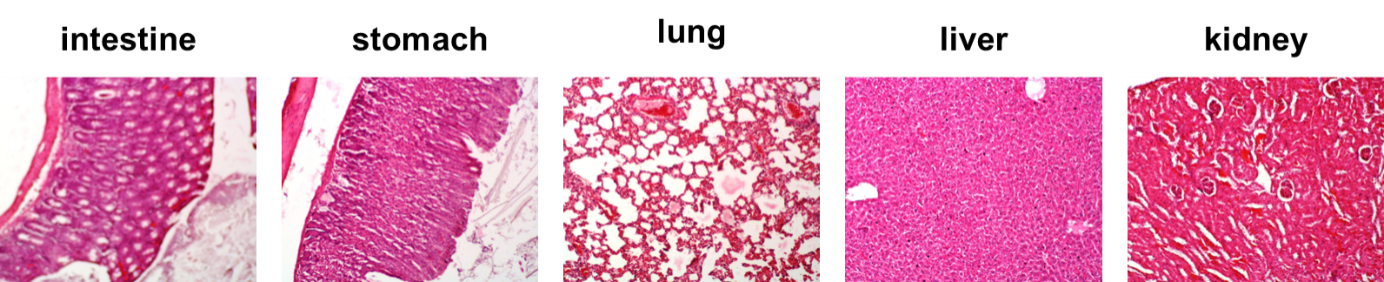
**

**Supplementary Figure 7.** Histopathology inspection of major organs of the mice treated with diclofenac, 16 days post-induction of the xenograft LS174T tumor (Hematoxylin and Eosin staining 40x). The treated mice received three intraperitoneal injections of diclofenac (40 mg/kg) 7, 9, and 11 days after injection of the tumor cells once a day.

**Supplementary Table 1.** Summary of the radiobiological parameters shown in Figure 5 A-D.

| **Cell line** | **D_50_ (Gy)^a^** | **SER^b^** | **α (Gy^−1^)^c^** | **β (Gy^−1^)^c^** |
| --- | --- | --- | --- | --- |
| **LS174T** |  |  |  |  |
| 0.0 mM Dic | 2.75 | 1.00 | 0.15 | 0.04 |
| 0.1 mM Dic | 1.85 | **1.48** | 0.21 | 0.09 |
| **LoVo** |  |  |  |  |
| 0.0 mM Dic | 2.96 | 1.00 | 0.09 | 0.05 |
| 0.1 mM Dic | 2.00 | **1.48** | 0.14 | 0.10 |
| **A549** |  |  |  |  |
| 0.0 mM Dic | 6.83 | 1.00 | 0.06 | 0.01 |
| 0.1 mM Dic | 6.58 | 1.04 | 0.10 | 0.001 |
| **MDA-MB-231** |  |  |  |  |
| 0.0 mM Dic | 3.77 | 1.00 | 0.03 | 0.04 |
| 0.1 mM Dic | 3.59 | 1.05 | 0.01 | 0.05 |

^a^ D_50_: dose (Gy) required for 50% inactivation of a tumor cell population.

^b^ SER: Sensitizing enhancement ratio = D_50_ (control)/D_50_ (drug treatment). A SER greater than 1.20 indicates a radio-sensitization (indicated in bold).

^c^ α and β values were derived from the linear quadratic equation f= exp(−α*x − β*x^2^).

Dic: Diclofenac

**Supplementary Methods**

**Cellular Reactive Oxygen Species (ROS) Assay**

**2`7`-dichlorofluorescin diacetate (DCFDA) Cellular ROS Detection Assay Kit (ab113851, Abcam, Cambridge, UK) was used to measure intracellular reactive oxygen species (ROS) in LS174T and A549 tumor cells by flow cytometry on a BD FACSCalibur**™ **instrument (BD Biosciences, Heidelberg, Germany) following the manufacturer`s protocol.**

**Pathology Evaluation**

An intraperitoneal injection of sodium pentobarbital (800 mg/kg body weight) was used to euthanize the animals 16 days after the LS174T cell injection. An examination of the peritoneal membrane and cavity was performed to detect macroscopic lesions. The main organs, including the stomach, kidneys, liver, intestine, and lungs, were removed and then transferred for formalin fixation and paraffin embedding. Following the preparation of the tissue sections, the staining with hematoxylin & eosin was performed. Careful examination and observation of the prepared slides of the tissue sections were conducted.
