# Supplementary material for: The radiation- and chemo-sensitizing capacity of diclofenac can be predicted by a decreased lactate metabolism and stress response
Source: Radiat Oncol. 2024 Jan 16;19:7. doi: 10.1186/s13014-024-02399-5 (PMC10790495; doi:10.1186/s13014-024-02399-5)

Figure 1 E: LS174T

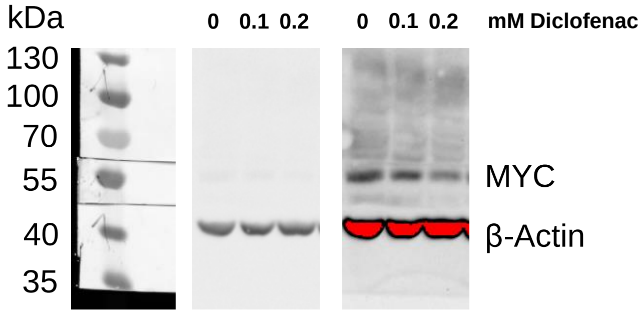

Figure 1 F: LoVo

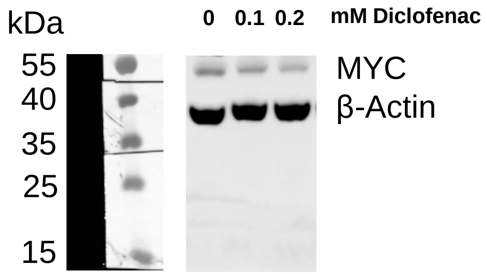

Figure 1 G: A549

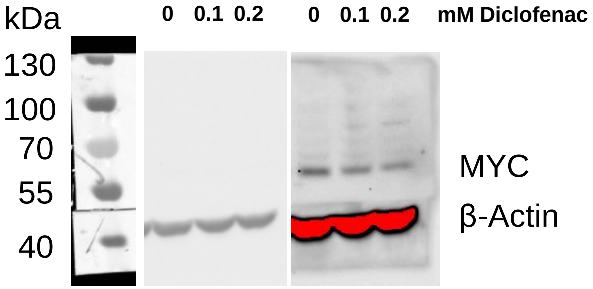

Figure 2 B: LS174T

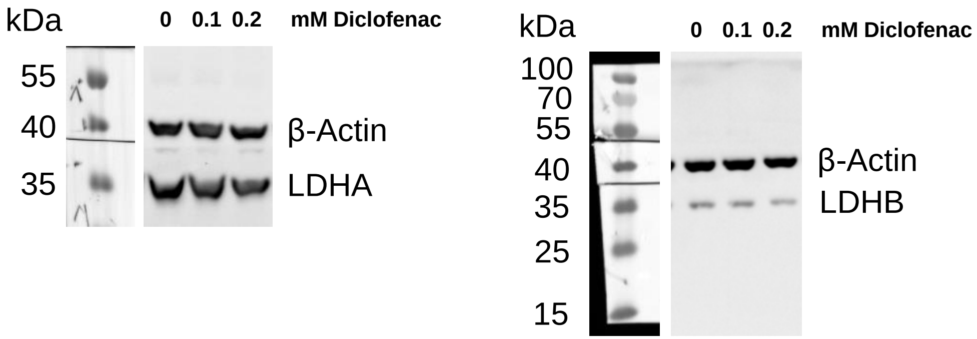

Figure 2 D: LoVo

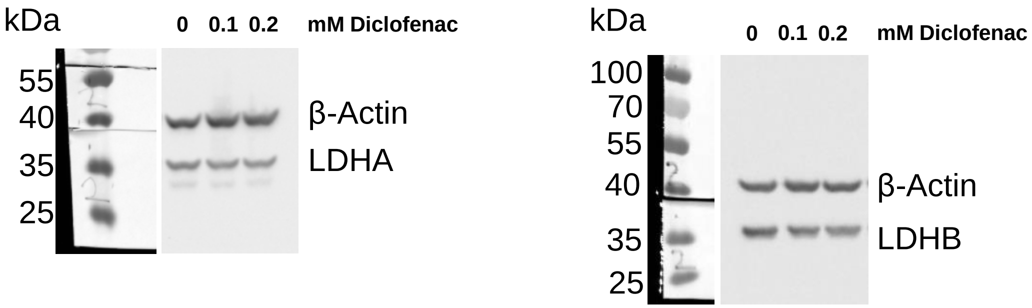

Figure 2 F: A549

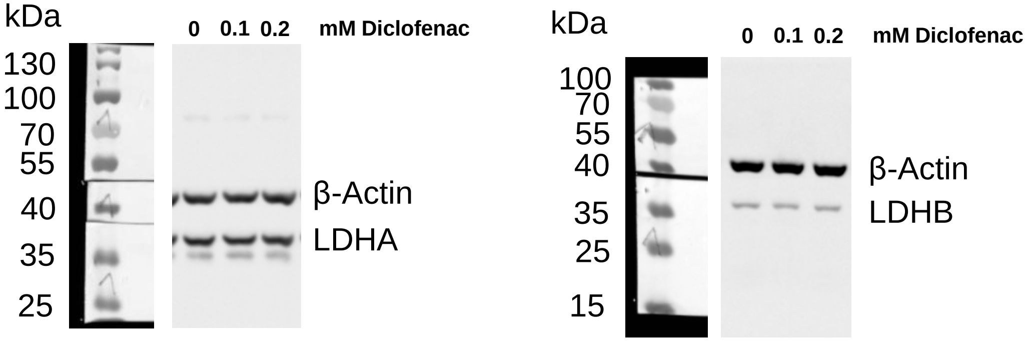

Figure 2 H: MDA-MB-231

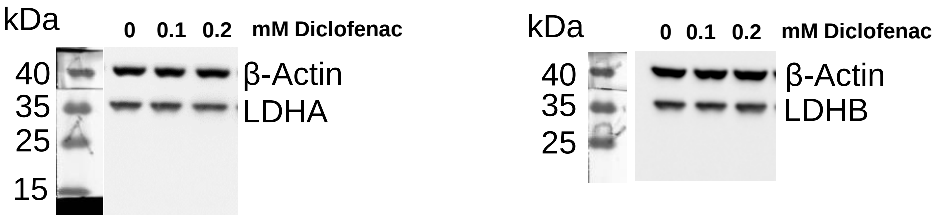

Figure 3 A: LS174T

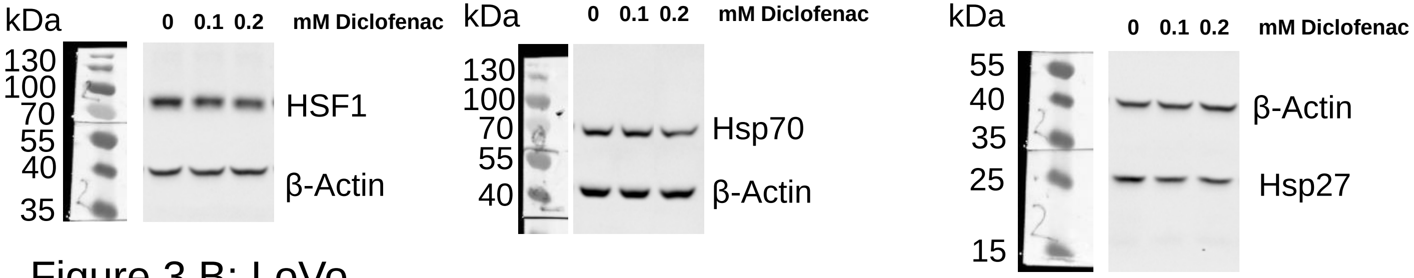

Figure 3 B: LoVo

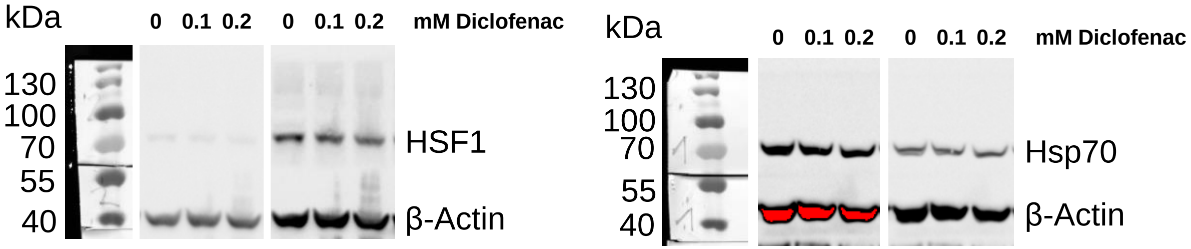

Figure 3 C: A549

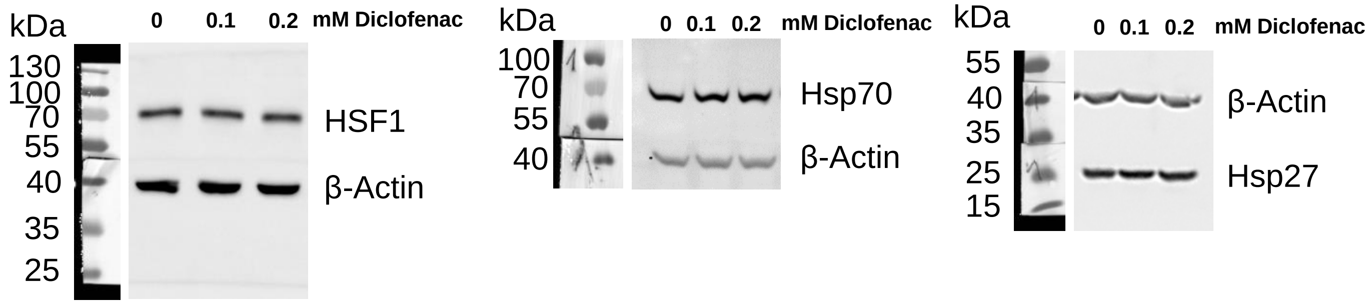

Figure 3 D: MDA-MB-231

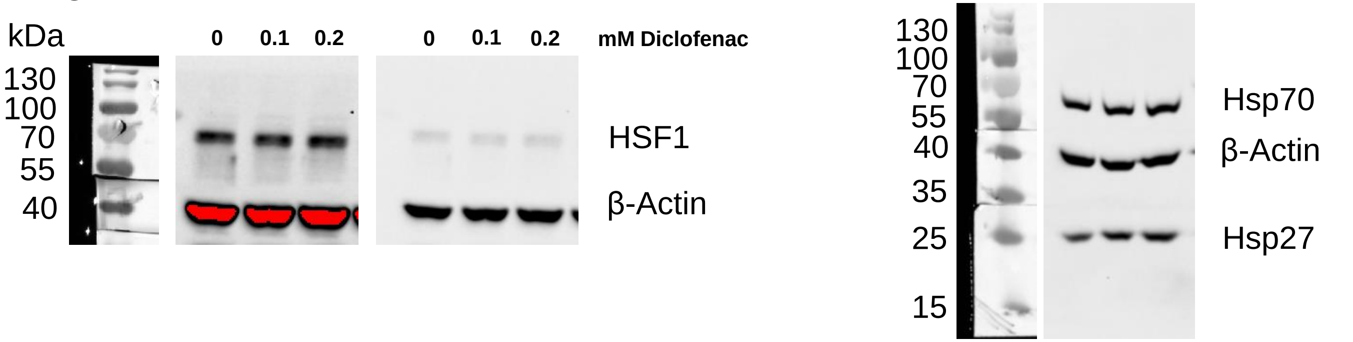

Figure S2 B: COLO-357

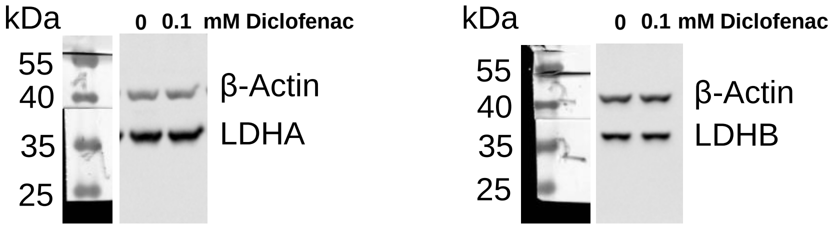

Figure S3: COLO-357

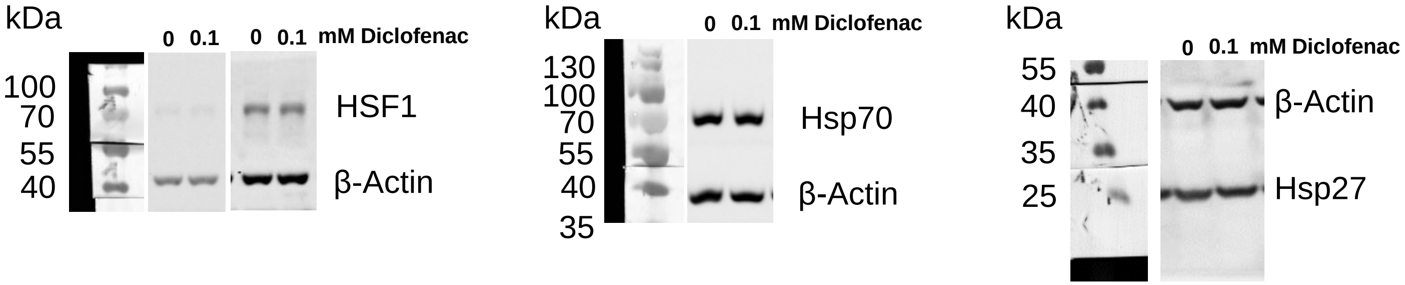

Supplement: Supplementary file 2 — Supplementary Material 2 [file 13014_2024_2399_MOESM2_ESM.pdf]
